# Bacterial Heavy-Metal and Antibiotic Resistance Genes in a Copper Tailing Dam Area in Northern China

**DOI:** 10.3389/fmicb.2019.01916

**Published:** 2019-08-20

**Authors:** Jianwen Chen, Junjian Li, Hong Zhang, Wei Shi, Yong Liu

**Affiliations:** ^1^Institute of Loess Plateau, Shanxi University, Taiyuan, China; ^2^School of Environment and Resources, Shanxi University, Taiyuan, China

**Keywords:** metal resistance genes, antibiotic resistance genes, mobile genetic element, co-occurrence, metal pollution, risk index, copper tailing

## Abstract

Heavy metal resistance genes (MRGs) and antibiotic resistance genes (ARGs) in bacteria can respond to the inducement of heavy metals. However, the co-occurrence of MRGs and ARGs in the long-term heavy metal contaminated area is still poorly understood. Here, we investigated the relationship between the abundance of soil bacteria MRGs, ARGs and heavy metal pollution in a copper tailing dam area of northern China. We found that *ars*C and *ere*A genes coding for resistance mechanisms to arsenic and to macrolides, respectively, are the most abundant MRG and ARG in the study area. The abundance of MRGs is positively correlated with cadmium (Cd) concentration, and this indicates the importance of Cd in the selection of MRGs. The network analysis results show that *sul*II and MRGs co-occur and *cop*B occur with ARGs, which suggests that MRGs and ARGs can be co-selected in the soil contaminated by heavy metal. The network analysis also reveals the co-occurrence of Cd and MRGs, and thus heavy metal with a high ‘toxic-response’ factor can be used as the indicator of MRGs. This study improves the understanding of the relationship between bacterial resistance and multi-metal contamination, and underlies the exploration of the adaptive mechanism of microbes in the multi-metal contaminated environment.

## Introduction

The rapid industrialization has led to a series of ecological and environmental problems (Sawut et al., 2018). One of the most urgent issues is heavy metal contamination that is harmful to both ecosystem functions and human health. Heavy metals are widely distributed in almost all types of soils (Zhao et al., 2012), sediment (Gati et al., 2016), and water bodies (Tang et al., 2014). Some heavy metals are essential micronutrients for several cellular functions and components of biological macromolecules [for example, zinc (Zn) is an important component of DNA-polymerases] (Seiler and Berendonk, 2012), but can be toxic when accumulated to a certain concentration (for example, the excessive uptake of Zn leads to zinc-induced copper deficiency) (Fosmire, 1990; Zhao et al., 2012). In recent years, industrial (e.g., mining) and agricultural (e.g., land application of metal contained fertilizers) activities contribute to heavy metal accumulation (Charlesworth et al., 2011; Chen et al., 2015; Li L.G. et al., 2017).

Environmental compartments are subject to anthropogenic pressures (Berendonk et al., 2015). As a vital component of terrestrial ecosystems, soil microbes play a significant role in the material and energy cycle. However, microbial communities are highly sensitive to environmental changes (Kandeler et al., 2000; Blakely et al., 2002; Kelly et al., 2003; Epelde et al., 2015). The excessive heavy metals found in the soil may impose selection pressures on soil microbes (Berendonk et al., 2015) and even change the diversity of soil microbial communities (Kandeler et al., 1996, 2000; Kelly et al., 2003; Epelde et al., 2015). To cope with these situations, an effective strategy for microorganisms is to evolve a system based on biochemical and genetically encoded mechanisms (Aka and Babalola, 2017). This has been found in many bacteria strains isolated from different heavy metal polluted scenarios (Zafar et al., 2007; Sabry et al., 2010; Rehman and Anjum, 2011; Muñoz et al., 2012). Bacteria can thus be used for the remediation of heavy metal polluted areas (Aka and Babalola, 2017; Chen et al., 2018). Nevertheless, present efforts of heavy metal resistance are mainly centered on single isolated strains (Zafar et al., 2007; Sabry et al., 2010; Rehman and Anjum, 2011; Muñoz et al., 2012). A better understanding of the distribution of resistance in heavy metal contaminated area, especially the long-term polluted sites, is critical to optimize such remediation schemes.

Antibiotic resistant genes (ARGs) have been reported as a new pollutant by the World Health Organization because of their emerging prevalence and wide distribution (World Health Organization [WHO], 2014). This is a threat to public health (Rodriguez-Mozaz et al., 2015). The increasing ARGs have been recognized as a consequence of the massive use of antibiotics in therapeutics and agriculture (Levy and Marshall, 2004; Huerta et al., 2013). However, more evidence shows that the dissemination of ARGs can also be influenced by heavy metal contamination (Pruden et al., 2006; Knapp et al., 2017; Tan et al., 2018). As early as in the 1970s, it was found that heavy metal resistance and antibiotic resistance can be selected simultaneously in the heavy metal contaminant ecosystem (Timoney et al., 1978). These phenomena can be interpreted on the molecular level as co-selection (two or more genetically linked resistance genes) or cross-selection (single genetic element provides tolerance to more than one antimicrobial agents) (Baker-Austin et al., 2006; Seiler and Berendonk, 2012). The co-selection of heavy metal resistance genes (MRGs) and ARGs have been reported in agriculture (Hu et al., 2016), animal husbandry (Zhu et al., 2013), wastewater treatment system (Cesare et al., 2016), and sediment (Wright et al., 2010). However, bacterial communities are shaped by a complex array of evolutionary, ecological, and environmental factors (Berendonk et al., 2015). Patterns of MRGs and ARGs in the area of long-term heavy metal contamination are still poorly studied.

Zhongtiaoshan Copper Mine is the largest non-coal underground mine in northern China (Liu et al., 2018). A huge tailing dam was built as a result of the long-term mining processes. The body of the dam was formed by large amounts of waste residue containing multiple heavy metals. As a result, the surrounding area of the dam is contaminated by heavy metals through wind or seep water.

The purpose of this study is to assess the heavy metal and antibiotic resistance in the downstream region of the tailing dam from the following aspects: (1) what the heavy metal contamination level is in the region, (2) what the main MRGs types are and how they are distributed in this region, and (3) what the co-occurrence patterns of MRGs and ARGs are in this region. The objective of this study is to reveal the occurrence of MRGs and ARGs and the relationships between heavy metal and MRGs/ARGs in this copper tailing dam area.

## Materials and Methods

### Soil Sampling

**FIGURE 1:**
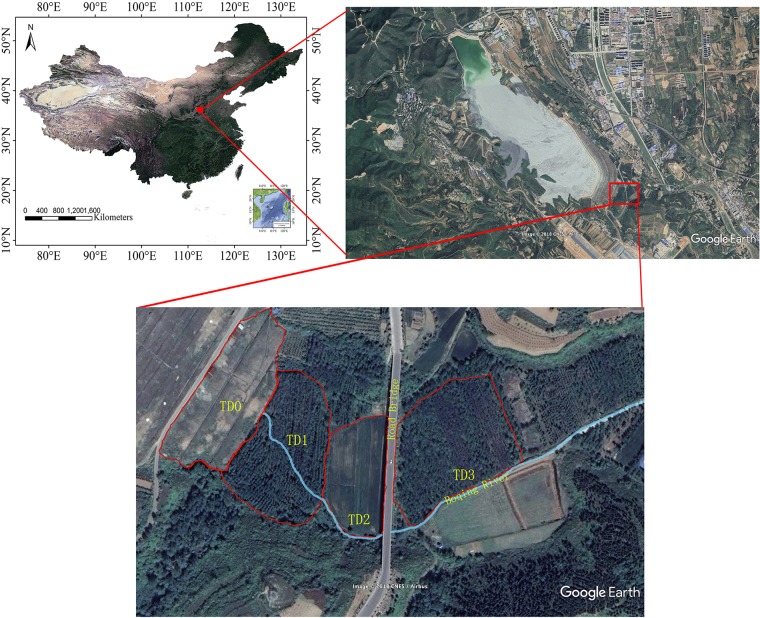
Locations of sampling sites around the Shibahe tailings dam area in Shanxi Province, China.

The study area (35°15′N, 111°39′E) is located in a copper tailing dam area in the southern part of Shanxi Province, China. The climate is monsoonal climate, with average annual temperature 14°C, mean annual rainfall 780 mm and annual frost-free period more than 200 days (Liu et al., 2018). The tailing dam has been used since 1972 (Figure 1). At the bottom of the tailing dam, a stream forms due to the seeping water. Four sampling sites TD0, TD1, TD2, and TD3 were selected along the stream in this study. TD0 site is on the top of the tailing dam and is covered by herbs. TD1 site is at the bottom of the dam and has been associated with poplar plantation of about 15 years. TD2 site, adjacent to TD1 site, is farmland. TD3 site is the most far away from the dam and has been associated with poplar plantation of about 10 years.

Sampling was carried out in June 2016, just after the wheat harvest. The upper 20 cm of soil was collected from three random locations at the TD0 site and nine random locations at each of the other three sites. All of the soil samples were sieved, thoroughly homogenized, and divided into two portions. One portion was stored at −80°C for molecular analysis, and the other was air-dried for chemical analysis. Soil pH in ddH_2_O was measured at a soil:solution ratio of 1:2.5 (w:v). Soil total carbon (TC), total nitrogen (TN), and total sulfur (TS) were determined by vario macro cube elemental analyzer (vario macro cube, elementar, Germany) (Table 1).

**TABLE 1:** Soil physicochemical characteristics and heavy metal content (mg kg^–1^ dry soil) at each sampling site.

**Sampling site**	**TD0**	**TD1**	**TD2**	**TD3**	**Background value**
pH	8.00 ± 0.02^ab^	7.76 ± 0.20^c^	8.04 ± 0.21^a^	7.80 ± 0.11^bc^	–
TN (g/kg)	0.58 ± 0.03^c^	0.89 ± 0.16^b^	1.12 ± 0.06^a^	0.89 ± 0.07^b^	–
TC (g/kg)	12.28 ± 0.26^c^	16.20 ± 1.27^a^	13.95 ± 0.46^b^	15.86 ± 1.52^a^	–
TS (g/kg)	1.51 ± 0.71^a^	0.73 ± 0.10^b^	0.59 ± 0.02^b^	0.68 ± 0.11^b^	–
As	6.94 ± 2.42^b^	12.28 ± 4.32^a^	7.15 ± 3.44^b^	10.52 ± 3.96^ab^	9.1
Cd	0.17 ± 0.08^c^	1.25 ± 0.66^a^	0.54 ± 0.34^bc^	0.76 ± 0.25^ab^	0.1
Cr	47.24 ± 20.04^b^	381.91 ± 176.75^a^	371.90 ± 169.15^a^	339.05 ± 174.71^a^	55.3
Cu	338.00 ± 184.21^a^	323.28 ± 157.59^a^	122.10 ± 36.15^b^	157.91 ± 36.37^b^	22.9
Ni	34.17 ± 7.63^a^	79.51 ± 55.13^a^	77.89 ± 44.53^a^	101.79 ± 67.16^a^	29.9
Pb	756.76 ± 221.91^a^	1227.24 ± 360.65^a^	983.42 ± 292.03^a^	1098.53 ± 349.17^a^	14.7
Zn	33.43 ± 18.91^b^	77.17 ± 36.37^a^	89.23 ± 23.84^a^	64.67 ± 16.73^ab^	63.5

*Data are means ± standard deviation. The different letters indicate that the means are significantly different among soils (*P* < 0.05) with Duncan test. TN, TC, and TS represent soil total nitrogen, soil total carbon and soil total sulfur, respectively.*

### Heavy Metal Content Analysis

Soil samples were digested using the HNO_3_- HF-H_2_O_2_ method (Raven and Loeppert, 1997) in a microwave digesting apparatus (CEM MARs XPRSS, United States). The digested samples were diluted with deionized water to 50 mL. The concentrations of heavy metals (As, Cr, Cd, Cu, Ni, Pb, and Zn) in the solutions were determined by inductively coupled plasma-optical emission spectrometry (ICP-OES, iCAP 6000, Thermo Fisher, United Kingdom). A mixed standard solution including all the heavy metals and reagent blanks were carried out through digestion and analyzed as part of the quality control protocol. Results were adopted when the measured concentrations in the reference materials were within one standard deviation of the certified values (Li et al., 2012).

### Ecology Risk Analysis

The potential ecological risk index (*RI*) is used to characterize the toxicity level of heavy metals. *RI* is the potential ecological risk caused by overall contamination (Hakanson, 1980), which covers a variety of research domains (e.g., biological toxicology, environmental chemistry, and ecology) (Zhai et al., 2014). *RI* can be used to evaluate the comprehensive ecological risks caused by a single pollutant and the overall risk from various pollutants. The assessment methods of *RI* are shown as follows:

$$C_{f}^{i}=\frac{C_{D}^{i}}{C_{R}^{i}}$$

$$R\,I=\sum_{i}^{n}E_{r}^{i}=\sum_{i}^{n}\left(T_{r}^{i}\times C_{f}^{i}\right)$$

Where $C_{f}^{i}$ is the single heavy metal pollution index, $C_{D}^{i}$ is the concentration of individual heavy metal in samples, $C_{R}^{i}$ is the reference value of heavy metal and usually uses the soil background value, $E_{r}^{i}$ is the monomial potential ecological risk factor of individual heavy metal, and $T_{r}^{i}$ is the heavy metal toxic response factor. The response values for each heavy metal are in the order of Zn = 1 < Cr = 2 < Cu = Pb = Ni = 5 < As = 10 < Cd = 30 (Hakanson, 1980).

### DNA Extraction and Quantitative Real Time PCR

Total genomic DNA was extracted from 0.25 g fresh soils using the Qiagen DNeasy PowerSoil Kit (Qiagen, Carlsbad, CA, United States) according to the manufacturer’s instructions. DNA concentration and purity were assessed by Infinite 200 PRO (TECAN, Sweden) and the DNA concentration of each sample was adjusted to yield a concentration of 10 ng μL^–1^. DNA extractions were stored at −20°C for further analysis.

The heavy MRGs were selected according to the findings of Li L.G. et al. (2017) that 9 MRGs types have distinct preference for ARG types as their closest genetic neighbor, and we also selected to test the antibiotic resistance genes that are widely detected in many long-term contaminated fields in China (Hu et al., 2016, 2017). Here, we quantified the absolute abundances of 13 MRGs, 28 ARGs, and 2 mobile genes elements (MGEs) through quantitative real-time PCR (qPCR). The MRGs include 5 copper resistance genes (*cop*A, *cop*B, *pco*A, *pco*C, and *pco*D), 3 multiple heavy MRGs (*czc*A, *czc*C, and *czc*D), 2 arsenic resistance genes (*ars*B and *ars*C) and 3 resistance genes (*ncc*A, *pbr*T, and *chr*B to nickel, lead, chromium, respectively). The ARGs include 15 tetracycline resistance genes (*tet*A, *tet*C, *tet*E, *tet*K, *tet*L, *tet*A/P, *tet*G, *tet*M, *tet*O, *tet*Q, *tet*S, *tet*T, *tet*W, *tet*B/P, and *tet*X), 4 β-lactam resistance genes (*bla*_*CTX–M*_, *bla*_*TEM*_, *bla*_*SHV*,_ and *bla*_*ampC*_), 3 sulfonamide resistance genes (*sul*I, *sul*II, and *sul*III), 3 quinolone resistance genes (*qnr*A, *qnr*B, and *qnr*S), and 3 macrolides resistance genes (*ere*A, *ere*B, and *mph*A). The MGEs include an integrase gene of class 1 integrons (*int*I1) and a transposon-transposase gene (*tnp*A).

The qPCR analysis was conducted using the iCycler iQ5 thermocycler (Bio-Rad, Hercules, CA, United States). Each sample was amplified in triplicate with 10 μL reaction mixture that consisted of 5 μL of SYBR Premix Ex Taq^TM^ (Takara Bio, Inc., Shiga, Japan), 0.5 μL of each primer (10 mM), 3 μL of diluted template DNA, and microbial DNA-free water. The primers and thermal cycling conditions of MRGs, ARGs and MGEs are described in Supplementary Table S1. A negative control was included in each run. Melting curve analysis was performed at the end of each qPCR run to check the specificity of the amplicons.

### Data Analysis

The data were square root- or log-transformed to improve normality and reduce heteroscedasticity. MRGs and ARGs data were converted to relative abundance (normalized per 16S rRNA genes) because the bacterial population size has not been taken into account for the non-normalized data. The 16S abundance rRNA genes were used to assess the size of the bacterial population. Normalizing MRGs or ARGs count to 16S rRNA genes presents an approximate proportion of bacteria that carry the corresponding genes.

One-way analysis of variance (ANOVA) was used to assess the differences of heavy metals, *RI*, 16S rRNA genes, MRGs, ARGs and MGEs among the study sites. Spearman linear correlation analysis was conducted to determine whether there were significant correlations between heavy metals, *RI*, MRGs, ARGs, and MGEs. Network analysis was used to explore the co-occurrence patterns of *RI*, MRGs, and ARGs.

ANOVA and Spearman linear correlation analysis were conducted in SPSS version 13.0 and R software version 3.4.4. Network analysis was performed and visualized with Gephi version 0.9.2.

## Results

### Physicochemical Characteristics and Risk Assessment

Soil total carbon (TC) and total nitrogen (TN) at the TD0 site are significantly lower than those at the other three sites, while the TS shows the opposite. Soil pH is significantly higher at the TD2 site than at the TD1 and TD3 sites (Table 1). There is no difference in the concentrations of Ni and Pb among the four sites; however, the concentrations of other metals (As, Cd, Cr, Cu, and Zn) are significantly different among the four sites (*P* < 0.05, Table 1). The concentrations of all the metals measured are highest at the TD1 site except the concentrations of Cu, Ni, and Zn that are highest in TD0, TD3, and TD2, respectively. The analysis of the overall risk shows that *RI* is significantly higher at the TD1 site than at the other three sites (*P* < 0.05, Table 2), while the TD0 site represents the lowest potential ecological risk of all the sampling sites.

**TABLE 2:** Potential ecology risk index (*RI*) at each sampling site.

**Sampling site**	**TD0**	**TD1**	**TD2**	**TD3**	**Contribute to *RI***
*RI*-As	7.63 ± 2.66^b^	13.5 ± 4.74^a^	7.86 ± 3.79^b^	11.56 ± 4.35^ab^	1.63%
*RI*-Cd	49.00 ± 23.73^c^	367.91 ± 192.79^a^	159.13 ± 100.72^bc^	223.98 ± 73.14^ab^	32.08%
*RI*-Cr	1.71 ± 0.72^b^	13.81 ± 6.39^a^	13.45 ± 6.12^a^	12.26 ± 6.32^a^	1.65%
*RI*-Cu	73.80 ± 40.22^a^	70.59 ± 34.41^a^	26.66 ± 7.89^b^	34.48 ± 7.94^b^	8.24%
*RI*-Ni	2.29 ± 0.51^a^	5.32 ± 3.69^a^	5.21 ± 2.98^a^	6.81 ± 4.49^a^	0.79%
*RI*-Pb	257.40 ± 75.48^a^	417.43 ± 122.67^a^	334.50 ± 99.33^a^	373.65 ± 118.77^a^	55.45%
*RI*-Zn	0.53 ± 0.30^b^	1.22 ± 0.57^a^	1.41 ± 0.38^a^	1.02 ± 0.26^ab^	0.17%
*RI*	392.35 ± 37.34^c^	889.78 ± 278.7^a^	548.21 ± 119.19^bc^	663.76 ± 148.39^b^	–

*Data are means ± standard deviation. The different letters indicate that the means are significantly different among soils (*P* < 0.05) with Duncan test. Contribute to *RI* means the percentage of the single metal ecological risk to the overall risk.*

### Absolute Abundance of 16S rRNA Genes, MRGs, ARGs, and MGEs

**FIGURE 2:**
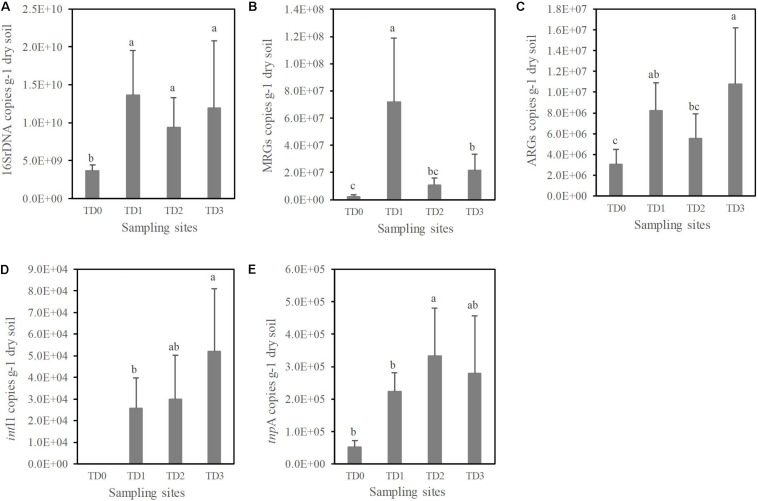
The abundances of 16S rDNA **(A)**, total detected metal resistance genes (MRGs) **(B)**, total detected antibiotic resistance genes (ARGs) **(C)** and mobile gene elements (*int*I1 and *tnp*A) **(D,E)** at each sampling site. TD0 site is on the tailing dam and covered with annual herbs. TD1 site with about 15 years of poplar plantation is at the bottom of the dam. TD2 site is farmland and adjacent to TD1 site. TD3 site with about 10 years of poplar plantation is the farthest away from the dam. Columns show the means of replicates within the sampling sites, and vertical bars show standard error. Different letters indicate that the means are significantly different among sampling sites with Duncan test (*P* < 0.05).

Quantitative PCR was performed to analyze the abundances of total bacteria (by targeting the bacterial 16S rRNA genes), MRGs, ARGs, and MGEs. The abundance of 16S rRNA genes at the TD0 site is significantly lower than at the other sites (*P* < 0.01, Figure 2A). A total of 10 MRGs, 17 ARGs, and 2 MGEs (out of 13 MRGs, 28 ARGs, and 2 MGEs targeted) are detected in this study. The lowest abundances of MRGs and ARGs are found at the TD0 site (Figures 2B,C). The highest abundance of MRGs is found at the TD1 site, while the highest abundance of ARGs is found at the TD3 site. There is a significant difference for MGEs among all the sampling sites (*P* < 0.05). The MGEs shows the lowest level at the TD0 site, and it has not been detected for the *int*I1 gene at the TD0 site (Figures 2D,E).

### The Distribution Patterns of MRGs

**FIGURE 3:**
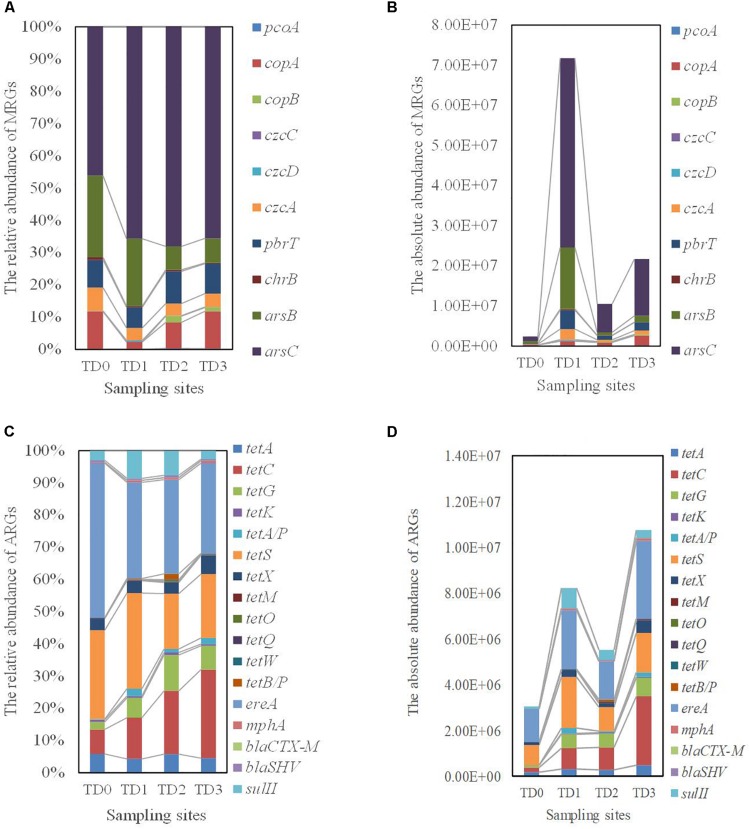
The distribution of Metal resistance genes (MRGs) and antibiotic resistance genes (ARGs) detected at each sampling site. TD0 site is on the tailing dam and covered with annual herbs. TD1 site with about 15 years of poplar plantation is at the bottom of the dam. TD2 site is farmland and adjacent to TD1 site. TD3 site with about 10 years of poplar plantation is the farthest away from the dam. **(A)** The distribution of relative abundance of MRGs. **(B)** The distribution of absolute abundance of MRGs. **(C)** The distribution of relative abundance of ARGs. **(D)** The distribution of absolute abundance of ARGs.

Of all the tested MRGs genes, *ars*B, *ars*C, *pbr*T, *czc*A, and *cop*A genes are major components at all the sampling sites. In particular, the *ars*C gene (coding for resistance mechanism to arsenic) accounts for nearly 46.13, 65.65, 68.48, and 65.52% of the total MRGs at the TD0, TD1, TD2, and TD3 site, respectively (Figure 3A). *cop*B genes have not been detected at the TD0 and TD1 sites, and *czc*C genes have only been detected at the TD1 site. In accordance with the sampling along the seeping stream, an increasing trend of all the copper resistance genes (*pco*A, *cop*A, and *cop*B) is observed, while all the other resistance genes is found at the highest abundance at the TD1 site and there are significant differences in these genes among all the sampling sites (*P* < 0.01) (Figure 3B and Supplementary Table S2).

### The Distribution Patterns of ARGs

The main genes of ARGs detected in this study are five tetracyclines resistance genes (*tet*A, *tet*C, *tet*G, *tet*S, and *tet*X), one sulfonamides resistance gene (*sul*II), and one macrolides resistances gene (*ere*A). They represent 98.08, 95.21, 94.07, and 95.66% of the total ARGs at the TD0, TD1, TD2, and TD3 sites, respectively (Figure 3C). The *ere*A gene has the largest distribution at each site, and it contributes to 48.04, 29.83, 29.22, and 28.00% of the total ARGs at each site, respectively (Figure 3C). When all ARGs are concerned, there are significant differences in *tet*C, *tetG*, *tet*A/P, *tet*X, *tet*M, *tet*O, *tet*Q, *tet*W, *tet*B/P, *mph*A, and *sul*II genes among the sampling sites (*P* < 0.05, Supplementary Table S3).

The ARGs patterns are more complex than MRGs. ARGs patterns can be classified as the following aspects: (1) there is no difference in the relative abundance of *tet*A, *tet*K, *tet*S, *ere*A, *bla*_*CTX–M*_, and *bla*_*SHV*_ genes among the sampling sites, and the highest abundance of these genes is found at the TD0 site apart from *tet*K and *bla*_*CTX–M*_ genes. (2) Similar to the majority of MRGs, the *sul*II gene is significantly more abundant at the TD1 site than at the other three sites. (3) The *tet*O, *tet*W, and *tet*B/P genes, which are not detected at the TD0 site, are significantly more abundant at the TD2 site than at the TD1 and TD3 sites. (4) There are significant differences in the relative abundance of the *tet*C, *tet*G, *tet*A/P, *tet*X, *tet*M, *tet*Q, and *mph*A genes among the sampling sites, with the highest abundance of those genes at the TD3 site (Figure 3D).

### Correlations Between the *RI*, MRGs, ARGs, and MGEs

**FIGURE 4:**
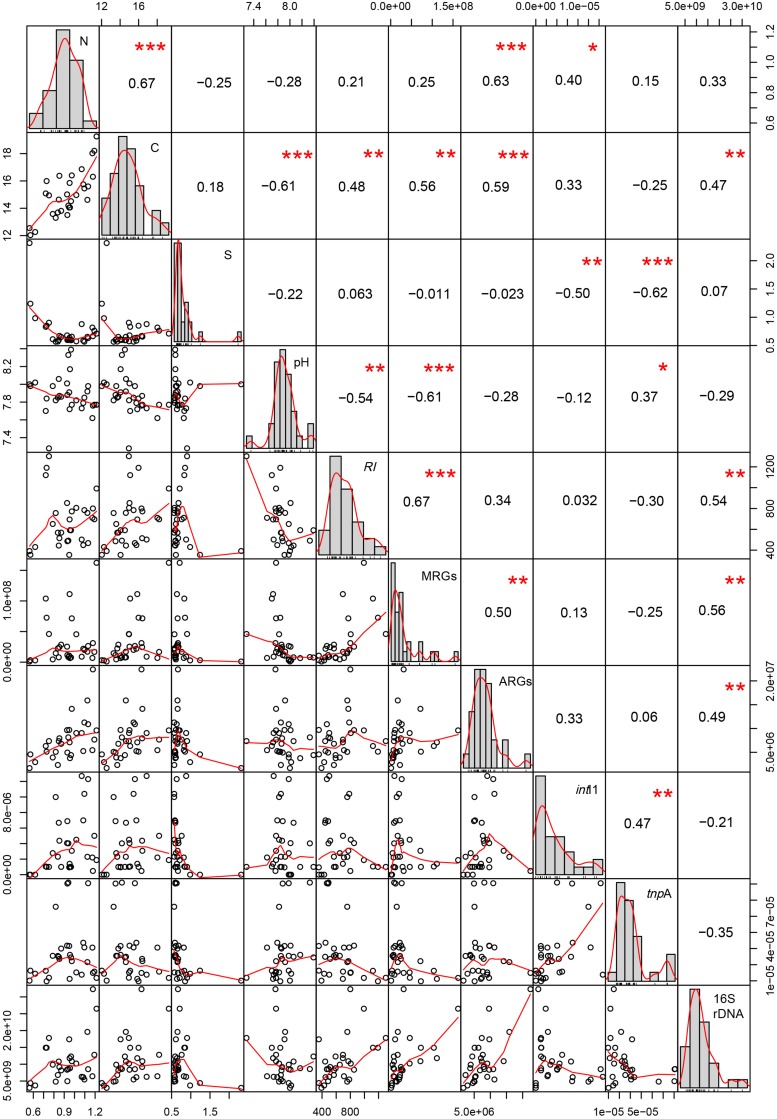
Correlations between soil physicochemical characteristics, *RI*, MRGs, ARGs, MGEs and 16S rDNA. ^∗∗∗^ Means that correlation is significant at the 0.001 level (two-tailed). ^∗∗^ Means that correlation is significant at the 0.01 level (two-tailed). ^∗^ Means that correlation is significant at the 0.05 level (two-tailed).

The potential *RI* is positively correlated with soil TC, the abundance of MRGs, and 16S rRNA genes (*P* < 0.01), and is negatively correlated with soil pH (*P* < 0.01, Figure 4). The abundance of MRGs is strongly positively correlated with TC, the abundance of ARGs and 16S rRNA genes (*P* < 0.01), and is negatively correlated with soil pH (*P* < 0.01). The abundance of ARGs is correlated with soil TN, TC, and the abundance of 16S rRNA genes. The abundance of *int*I1 gene is positively correlated with the abundance of the *tnp*A gene (*P* < 0.01) and TN (*P* < 0.05), and negatively correlated with soil TS. The abundance of the *tnp*A gene is positively correlated with soil pH (*P* < 0.05) and negatively correlated with TS (*P* < 0.01). The abundance of MGEs has no significant correlation with 16S rRNA genes (*P* > 0.05).

### Co-occurrence Patterns of MRGs and ARGs

**FIGURE 5:**
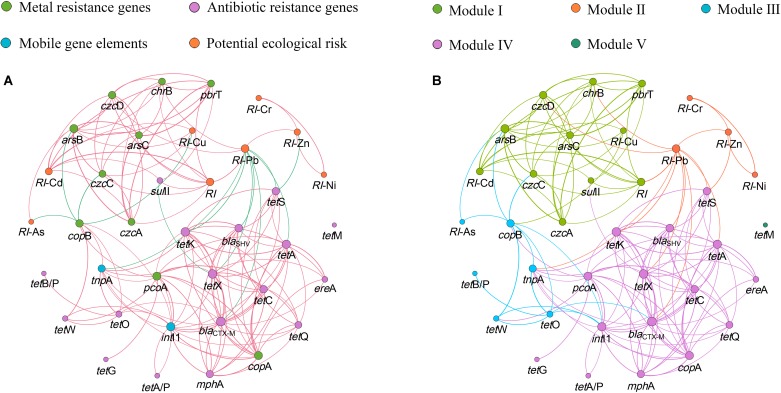
The network analysis showing the co-occurrence patterns among the detected MRGs, ARGs, MGEs and *RI*. **(A)** The nodes with different colors represent MRGs, ARGs, MGEs and *RI*, respectively. The red edges correspond to positive correlations, and the blue edges correspond to negative correlations. **(B)** The nodes with different colors represent different modularity classes, and the colors of edges correspond to the different modules. A connection stands for a strong (ρ > 0.6) and significant (*P* < 0.05) correlation. The size of each node is proportional to the number of connections.

Network analysis is conducted to explore the co-occurrence patterns among the relative abundance of MRGs, ARGs, and MGEs based on significant correlation analysis by Spearman correlation (*P* < 0.05). The resultant network consists of 37 nodes (8 *RI*s, 10 MRGs, 17 ARGs, and 2 MGEs) and 144 edges, and can be clearly separated into five modules (Figure 5). Genes in the same module may co-occur under the same environmental pressure. The majority of MRGs and ARGs coexist within their internal genes, while the *cop*A and *pco*A may co-exist with many ARGs, and *sul*II may co-exist with many MRGs. Genes co-existence with the MGEs indicate the mobility of these genes in the same module. The most densely connected node in each module is defined as ‘hubs,’ for example, in module III, *bla*_*ctx–M*_ may be the hub. The hubs can act as indicators to represent the quantity of other co-occurring genes in the same module.

## Discussion

### Heavy Metal Contamination and Bacterial Abundance

The *RI* results of heavy metals in the study area show that the TD1 site is suffering more ecological risk than the other three sites, and the TD0 site is at the lowest ecological risk level (Table 2). This distribution pattern results mainly from the accumulations of As, Cd, Cr, and Pb at the TD1 site (Table 1). This is probably related with the unique terrain conditions of this sampling site and the migration of heavy metals. There are two main sources of heavy metals for the TD1 site, of which one is the dissolved load with the leachate from the tailing dam (TD0) and the other is the sediment transported with the wash load (Merrington and Alloway, 1994). A similar accumulation pattern was observed in a similar area (Merrington and Alloway, 1994). The migration of metals in a dissoluble state in such an area can be achieved through the oxidation of metal sulfides, which aggravates the process of leaching when the solubility of heavy metal is increased in the form of metal sulfates (Simón et al., 2001).

The TS concentration is significantly higher at the TD0 site than at the other three sites (Table 1), and the soil at the TD0 site is alkalescence with the pH 8.00 ± 0.02 (Table 1). The high concentration of sulfur and the alkaline conditions indicate that the heavy metals exist in the form of metal sulfides at the TD0 site, which limits the migration of heavy metals to some extent. This interpretation is also supported by the significantly negative correlation between pH and *RI* (Figure 4). Thus, the transport of heavy metals from the TD0 site to the TD1 site is mainly the result of wash load. Evidence also shows that afforestation could effectively inhibit the oxidation of metal sulfides in the mining wasteland and reduce the production of acid wastewater and the release of heavy metals (Néel et al., 2003; Changul et al., 2010; Yang et al., 2010). After the accumulation of wash load, heavy metals can be fixed in the soil of the TD1 site under the influence of vegetation.

Although the highest heavy metal content is observed at the TD1 site, the TD1 site has the highest abundance of bacteria indicated by the abundance of the 16S rRNA genes (Figure 2A). Heavy metal contamination modifies the microbial diversity and biomass, because bacteria community is sensitive to heavy metals (Giller et al., 1998; Azarbad et al., 2015). Most studies have shown that high contamination of heavy metals can significantly decrease the soil bacterial biomass (Fließbach et al., 1994; Chander et al., 1995; Stefanowicz et al., 2010), but these studies focused on the short-term impacts of toxic pollutants. However, under a long-term polluted circumstance, Bourceret et al. (2015) found high richness and diversity of the bacterial community, and this is because edaphic parameters (nutrients like soil organic matter, soil available phosphorous, etc.) are increased simultaneously over the time of remediation. In this study, we also found a significant correlation between the soil bacterial abundance and TC (Figure 4).

### Heavy Metal and Metal Resistance

Heavy metals can form complex compounds, of which some are important for micronutrient physiological activities in all living microorganisms (Nies, 1999). However, most complex compounds are toxic at high concentrations (Nies, 1999). It is critical for microbial communities to develop the resistant systems (specific or unspecific) under long-term heavy metal stresses (Nies, 1999; Hemme et al., 2010; Chen et al., 2018). Such systems with the genetic basis of MRGs are inducible under adverse circumstances (Nies and Silver, 1995). The MRGs at the TD1 site are more abundant than at the other three sites (Figure 2B); this difference is also revealed by *RI* at the four sampling sites. This suggests that high *RI* would induce more bacterial resistance in the long-term contaminated area, which is also supported by the significant correlation between the abundance of MRGs and *RI* (*P* < 0.001) (Figure 4).

Soil copper in the form of divalent copper is dominant in our study site; both *cop*A and *pco*A function by the way of oxidizing Cu^+^ to Cu^2+^ for the purpose of detoxifying (Rensing and Grass, 2003). Therefore, both *pco*A and *cop*A genes are not significantly correlated with soil Cu contents (Supplementary Table S4). This relationship was also observed in sediment (Roosa et al., 2014). The *cop*B gene in TD2 and TD3 is associated with low Cu concentration (Table 1 and Supplementary Table S2), indicating that *cop*B system may be functioned in low Cu circumstance.

Among these MRGs at all the sampling sites, *ars*B and *ars*C genes are the main components and they have the similar distribution pattern with arsenic and *RI* (Tables 1, 2, Figure 3A, and Supplementary Table S2). Arsenic is positively correlated with the abundances of *ars*B and *ars*C genes (Supplementary Table S4). The significantly positive correlation between arsenic and *ars* gene was also found in the batch growth experiments (Poirel et al., 2013). Arsenic can induce the expression of *ars* genes (Tauriainen et al., 1997; Lópezmaury et al., 2003; Ordóñez et al., 2005), and *ars* genes may be suitable for exploring the microbial response to arsenic stress.

There are significant correlations between Cd and its target resistant genes (*czc*A, *czc*C, *czc*D). Meanwhile, Cd is also significantly correlated with *pbr*T, *chr*B, *ars*B, and *ars*C, respectively (*P* < 0.05) (Supplementary Table S4). This can be explained by the fact that different resistance genes may be located on the same unit of selection such as the same plasmid, transposon, or genome (Monchy et al., 2007). Van Houdt et al. (2009) reported that both Pb and Zn resistance increased because the transposon named *Tn*6048 proliferated under the Pb contaminated environment. Cd with a ‘toxic-response’ factor equal to 30 and Pb with a ‘toxic-response’ factor equal to 5 (Hakanson, 1980) contribute 32.08 and 55.45% to *RI*, respectively (Table 2). However, the abundance of MRGs is strongly correlated with Cd rather than Pb (Supplementary Table S4), and the network analysis reveals that MRGs and *RI*-Cd, not *RI*-Pb, were found in Module I (Figure 5). This indicates that heavy metal with high ‘toxic-response’ factor may be an indicator of the MRGs in the heavy metal contaminated area.

### Heavy Metal and the Co-occurrence of MRGs and ARGs

There are 17 ARGs detected in the copper tailing dam area (Supplementary Table S3), and ARGs exist in many metal contaminated circumstances (Hu et al., 2016, 2017). Correlations exist between metals and ARGs, but the trends are diversified (Knapp et al., 2017). In this study, significant correlations are observed between specific ARGs and heavy metals (Figure 5 and Supplementary Table S5). For example, Zn is significantly correlated with *sul*II. Ji et al. (2012) reported that Zn was positively correlated with *sul*I and *sul*III, rather than *sul*II in agriculture soil and manure samples. However, *sul*I and *sul*III are not detected in this study. The different samples may account for the distinct results. Previous studies have reported that Zn directly triggers the selection of tetracycline resistance genes including *tet*A, *tet*C, and *tet*G, genes functioning as an efflux pump system (Yamaguchi et al., 1990; Palm et al., 2008). We find that Zn is significantly correlated with *tet*W and *tet*B/P, genes encoding ribosomal protection proteins, which are detected in metal polluted areas (Berg et al., 2010; Knapp et al., 2011, 2017). This indicates that Zn may induce both the efflux pump system and ribosomal protection proteins.

The network analysis reveals strong correlation between *sul*II and MRGs in Module I. *pco*A and most of ARGs are detected in Module III (Figure 5), suggesting the possibility of the co-occurrence of MRGs and ARGs in soil microbe. The co-occurrence of MRGs and ARGs is verified by an IncA/C plasmid carrying *mer* operon (coding for resistance mechanism to mercury) and ARGs including *sul*II harbored in the *Aeromonas salmonicida* sub sp. *salmonicida* strains isolated from aquaculture facilities (McIntosh et al., 2008). *Int*I1 and *tnp*A are in the different modules, and show different correlations with each specific MRGs and ARGs (Figure 5). The similar correlations between MGEs and ARGs were observed in greenhouse soils with long-term dairy cattle and chicken manure (Li J. et al., 2017). Previous studies reported that the same ARGs or MRGs could be located in the same or different conjugative MGEs (Gillings et al., 2008; Herrick et al., 2014). Future studies are necessary to carry out detailed investigations of the horizontal transfer mechanism of MRGs and ARGs.

## Conclusion

This study selected a long-term heavy metal contaminated area, the Shibahe copper tailing dam area in Mountain Zhongtiaoshan of northern China, analyzed the levels of the contamination of multiple metals, and explored the relationship between soil microbial resistance and multi-metal contamination. Based on *in situ* monitoring, *RI* is adopted to evaluate the level of contamination. The abundance of soil bacteria is high at the high-*RI* site, and this is probably attributed to high soil nutrient and strong bacterial resistance. Both MRGs and ARGs exist in the multi-metal polluted soil. Of the MRGs, *ars*C coding for resistance mechanism to arsenic is at the highest abundance; *ere*A, which belongs to a deactivate system against macrolides, is the most abundant among the ARGs. The abundance of MRGs is positively correlated with Cd concentration, indicating that Cd plays a key role in the selection of MRGs. For ARGs, the abundances of *tet*W and *tet*B/P are significantly correlated with Zn concentration, which indicates that Zn may induce the antibiotic resistance of ribosome protection in such a long-term copper tailing dam area. The network analysis results show that *sul*II and MRGs co-occur and *cop*B occur with ARGs, and this suggests that MRGs and ARGs can be co-selected in the heavy metal contaminated soil. The network analysis also reveals the co-occurrence of Cd and MRGs, and accordingly heavy metal with a high ‘toxic-response’ factor can be used to indicate the occurrence of MRGs. In all, this study highlights the necessity to consider the contamination of multi heavy metals when assessing microbial resistance, and further improves the understanding of the relationship between bacterial resistance and multi-metal contamination. Meanwhile, our findings are alarming for the future evaluation of public health risk associated with heavy metal-induced selection of ARGs in multi-metal polluted environments.

## Author Contributions

JC wrote the main manuscript and prepared the figures. JL helped with the sampling design and prepared the data analytical methods. WS helped in the field work, sampling, and soil parameter achieving. HZ prepared the assessment method. YL was responsible for project administration and funding acquisition. HZ and YL provided many suggestions for the experimental design and implementation. All authors reviewed the manuscript.

## Conflict of Interest Statement

The authors declare that the research was conducted in the absence of any commercial or financial relationships that could be construed as a potential conflict of interest.
