# Supplementary material for: Bacterial Heavy-Metal and Antibiotic Resistance Genes in a Copper Tailing Dam Area in Northern China
Source: Front Microbiol. 2019 Aug 20;10:1916. doi: 10.3389/fmicb.2019.01916 (PMC6710345; doi:10.3389/fmicb.2019.01916)
Supplement: Supplementary file 1 [file Data_Sheet_1.PDF]

Supplemental Material

Bacteria Resistance to Metal Pollution in a Copper Tailing Dam Area in Northern China

Jianwen Chen[1], Junjian Li[1], Hong Zhang[2], Wei Shi[1], Yong Liu[1,]*

[1] *Institute of Loess Plateau, Shanxi University, Taiyuan, Shanxi, 030006, China*
[2] *School of Environment and Resources, Shanxi University, Taiyuan 030006, China*

TABLE S1 Target genes and primer sequences used in this study

	gene name	Function		Primer 5'-3' forward and reverse	Product size (bp)	Annealing temperature (℃)	Reference
MRGs	*cop*A	Cu translocating ATPase	F	CGG TCT CTA CGA ATA CCG CTT CAA	300	55	Bouskill et al., 2007
			R	GAA ATA GCT CAT TGC CGA GGC GTT			
MRGs	*cop*B	Cu binding protein	F	TTC CTG CTC GAC CAG TTG GAA TAC	450	58	Bouskill et al., 2007
			R	GGT TGG TCA ACA GGA TGT CGT ACT			
MRGs	*pco*A	multi-copper oxidase	F	ATA CCA TCT TCG CAC AAT CCA	194	60	Lee et al., 2002
			R	GCT GTT ATC CAT GCC TCC C			
MRGs	*pco*C	Periplasmic protein	F	TGT CGA TTT TAA ATA AAG CCA TTC TT	400	60	Lee et al., 2002
		periplasmic copper handling	R	TCA TAA TCA GGT CGT TCA TAA TAT TAC TTC			
MRGs	*pco*D	Periplasmic protein	F	GAT ATC TGA CTC CCT GGC	734	60	Lee et al., 2002
		periplasmic copper handling	R	CCG TAA AAT CAA AGG GCT			
MRGs	*czc*A	Co/Zn/Cd efflux protein	F	GTT CAC CTT GCT CTT CGC CAT GTT	320	56	Bouskill et al., 2007
			R	ACA GGT TGC GGA TGA AGG AGA TCA			
MRGs	*czc*C	Outer part of a Co/Zn/Cd	F	AGC CGY CAG TAT CCG GAT CTG AC	418	62	Roosa et al., 2014
		efflux protein (RND)	R	GTG GTC GCC GCC TGA TAG GT			
MRGs	*czc*D	Cation diffusion	F	TCA TCG CCG GTG CGA TCA TCA T	272	55	Roosa et al., 2014
		facilitator	R	TGT CAT TCA CGA CAT GAA CC			
MRGs	*ncc*A	Inner part of a Ni/Co efflux	F	TTY AGC CAG GTV ACS GTS ATY TT	532	63	Roosa et al., 2014
		protein (RND)	R	GCY GCR TCS GCR CGC ACC AGR TA			

	gene name	Function		Primer 5'-3' forward and reverse	Product size (bp)	Annealing temperature (℃)	Reference
MRGs	*pbr*T	Pb uptake protein	F	AGC GCG CCC AGG AGC GCA GCG TCT T	448	55	Roosa et al., 2014
			R	GGC TCG AAG CCG TCG AGR TA			
MRGs	*chr*B	Cr efflux protein	F	GTC GTT AGC TTG CCA ACA TC	450	57	Nie et al., 1990
			R	CGG AAA GCA AGA TGT CGA TCG			
MRGs	*ars*B	Arsenite efflux ATPase	F	GGT CTA TGC GCT GGA GCA ATT GAA	500	46	Bouskill et al., 2007
			R	TGC TGG GCA TGT TGT TCA TTA CCG			
MRGs	*ars*C	Arsenate reductase protein	F	GCA GCA TTC TTT CCG AAG CCA TGT	215	46	Bouskill et al., 2007
			R	TCG CAA ACG GTG ATG ACG ATG T			
ARGs	*tet*A	Tetracycline efflux	F	GCT ACA TCC TGC TTG CCT TC	210	55	Ng et al., 2001
			R	CAT AGA TCG CCG TGA AGA GG			
ARGs	*tet*C	Tetracycline efflux	F	CTT GAG AGC CTT CAA CCC AG	418	55	Ng et al., 2001
			R	ATG GTC GTC ATC TAC CTG CC			
ARGs	*tet*E	Tetracycline efflux	F	GTT ATT ACG GGA GTT TGT TGG	278	55	Ng et al., 2001
			R	AAT ACA ACA CCC ACA CTA CGC			
ARGs	*tet*G	Tetracycline efflux	F	GCT CGG TGG TAT CTC TGC TC	468	55	Ng et al., 2001
			R	AGC AAC AGA ATC GGG AAC AC			
ARGs	*tet*K	Tetracycline efflux	F	CGA AAA CAG ACT CGC CAA TC	169	55	Ng et al., 2001
			R	TCC ATA ATG AGG TGG GGC			
ARGs	*tet*L	Tetracycline efflux	F	TCG TTA GCG TGC TGT CAT TC	267	55	Ng et al., 2001
			R	GTA TCC CAC CAA TGT AGC CG			
ARGs	*tet*A/P	Tetracycline efflux	F	CTT GGA TTG CGG AAG AAG AG	676	55	Ng et al., 2001
			R	ATA TGC CCA TTT AAC CAC GC			

	gene name	Function		Primer 5'-3' forward and reverse	Product size (bp)	Annealing temperature (℃)	Reference
ARGs	*tet*S	protection	F	CAT AGA CAA GCC GTT GAC C	667	55	Ng et al., 2001
			R	ATG TTT TTG GAA CGC CAG AG			
ARGs	*tet*X	unkown	F	CAA TAA TTG GTG GTG GAC CC	468	58	Ng et al., 2001
			R	TTC TTA CCT TGG ACA TCC CG			
ARGs	*tetM*	protection	F	ACA GAA AGC TTA TTA TAT AAC	171	45	Aminov et al., 2001
			R	TGG CGT GTC TAT GAT GTT CAC			
ARGs	*tetO*	protection	F	ACG GAR AGT TTA TTG TAT ACC	171	45	Aminov et al., 2001
			R	TGG CGT ATC TAT AAT GTT GAC			
ARGs	*tet*Q	protection	F	AGA ATC TGC TGT TTG CCA GTG	169	55	Aminov et al., 2001
			R	CGG AGT GTC AAT GAT ATT GCA			
ARGs	*tet*T	protection	F	AAG GTT TAT TAT ATA AAA GTG	169	40	Aminov et al., 2001
			R	AGG TGT ATC TAT GAT ATT TAC			
ARGs	*tet*W	protection	F	GAG AGC CTG CTA TAT GCC AGC	168	60	Aminov et al., 2001
			R	GGG CGT ATC CAC AAT GTT AAC			
ARGs	*tet*B/P	protection	F	AAA ACT TAT TAT ATT ATA GTG	169	40	Aminov et al., 2001
			R	TGG AGT ATC AAT AAT ATT CAC			
ARGs	*sul*I	protection	F	CGC ACC GGA AAC ATC GCT GCA C	163	56	Pei et al., 2006
			R	TGA AGT TCC GCC GCA AGG CTC G			
ARGs	*sul*II	protection	F	TCC GGT GGA GGC CGG TAT ATG G	191	61	Pei et al., 2006
			R	CGG GAA TGC CAT CTG CCT TGA G			
ARGs	*sul*III	protection	F	TCC GTT CAG CGA ATT GGT GCA G	128	60	Pei et al., 2006
			R	TTC GTT CAC GCC TTA CAC CAG C			

	gene name	Function		Primer 5'-3' forward and reverse	Product size (bp)	Annealing temperature (℃)	Reference
ARGs	*qnr*A	unkown	F	TCA GCA AGA GGA TTT CTC A	516	50	Kehrenberg et al., 2006
			R	GGC AGC ACT ATG ACT CCC A			
ARGs	*qnr*B	unkown	F	TCG GCT GTC AGT TCT ATG ATC G	469	54	Kehrenberg et al., 2006
			R	TCC ATG AGC AAC GAT GCC T			
ARGs	*qnr*S	unkown	F	TGA TCT CAC CTT CAC CGC TTG	566	58	Kehrenberg et al., 2006
			R	GAA TCA GTT CTT GCT GCC AGG			
ARGs	*ere*A	deactivate	F	AAC ACC CTG AAC CCA AGG GAC G	420	52	Sutcliffe et al., 1996
			R	CTT CAC ATC CGG ATT CGC TCG			
ARGs	*ere*B	deactivate	F	AGA AAT GGA GGT TCA TAC TTA CCA	546	52	Sutcliffe et al., 1996
			R	CAT ATA ATC ATC ACC AAT GGC A			
ARGs	*mph*A	deactivate	F	AAC TGT ACG CAC TTG C	837	52	Sutcliffe et al., 1996
			R	GGT ACT CTT CGT TAC C			
ARGs	$bla_{\text{CTX-M}}$	deactivate	F	ATG TGC AGY ACC AGT AAR GT	593	50	Shahid, 2010
			R	TGG GTR AAR TAR GTS ACC AGA			
ARGs	bla_{TEM}	deactivate	F	KAC AAT AAC CCT GRT AAA TGC	936	58	Shahid, 2010
			R	AGT ATA TAT GAG TAA ACT TGG			
ARGs	bla_{SHV}	deactivate	F	TTT ATC GGC CYT CAC TCA AGG	930	58	Shahid, 2010
			R	GCT GCG GGC CGG ATA ACG			
ARGs	bla_{ampC}	deactivate	F	CCC CGC TTA TAG AGC AAC AA	634	58	Shahid, 2010
			R	TCA ATG GTC GAC TTC ACA CC			
MGE	*int*I1	integrase	F	CTG GAT TTC GAT CAC GGC ACG	473	60	Hardwick et al., 2010
			R	ACA TGC GTG TAA ATC ATC GTC G			

	gene name	Function		Primer 5'-3' forward and reverse	Product size (bp)	Annealing temperature (℃)	Reference
MGE	*tnp*A	transposase	F	CCG ATC ACG GAA AGC TCA AG	101	60	Han et al., 2016
			R	CCG ATC ACG GAA AGC TCA AG			

MRGs: metal resistance genes. ARGs: antibiotic resistance genes. MGE: mobile gene elements.

Reference

Aminov, R.I., Garrigues-Jeanjean, N., ., and Mackie, R.I. (2001). Molecular ecology of tetracycline resistance: development and validation of primers for detection of tetracycline resistance genes encoding ribosomal protection proteins. Applied & Environmental Microbiology 67(1), 22.

Bouskill, N.J., Barnhart, E.P., Galloway, T.S., Handy, R.D., Ford, T.E., 2007. Quantification of changing Pseudomonas aeruginosa sodA, htpX and mt gene abundance in response to trace metal toxicity: a potential in situ biomarker of environmental health. FEMS Microbiology Ecology 60, 276-286.

Han, X.M., Hu, H.W., Shi, X.Z., Wang, J.T., Han, L.L., Chen, D., et al. (2016). Impacts of reclaimed water irrigation on soil antibiotic resistome in urban parks of Victoria, Australia ☆. Environmental Pollution 211, 48-57.

Hardwick, S.A., Stokes, H.W., Sophia, F., Mark, T., and Gillings, M.R. (2010). Quantification of class 1 integron abundance in natural environments using real-time quantitative PCR. Fems Microbiology Letters 278(2), 207-212.

Kehrenberg, C., Friederichs, S., De Jong, A., Michael, G.B., and Schwarz, S. (2006). Identification of the plasmid-borne quinolone resistance gene qnrS in Salmonella enterica serovar Infantis. Journal of Antimicrobial Chemotherapy 58(1), 18.

Lee, S.M., Grass, G., Rensing, C., Barrett, S.R., Yates, C.J., Stoyanov, J.V., Brown, N.L., 2002. The Pco proteins are involved in periplasmic copper handling in Escherichia coli. Biochemical & Biophysical Research Communications 295, 616-620.

Ng, L.K., Martin, I., Alfa, M., and Mulvey, M. (2001). Multiplex PCR for the detection of tetracycline resistant genes. Mol Cell Probes 15(4), 209-215.

Nies, A., Nies, D.H., Silver, S., 1990. Nucleotide sequence and expression of a plasmid-encoded chromate resistance determinant from Alcaligenes eutrophus. Journal of Biological Chemistry 265, 5648-5653.

Pei, R., Kim, S.C., Carlson, K.H., and Pruden, A. (2006). Effect of River Landscape on the sediment concentrations of antibiotics and corresponding antibiotic resistance genes (ARG). Water Research 40(12), 2427-2435.

Roosa, S., Wattiez, R., Prygiel, E., Lesven, L., Billon, G., Gillan, D.C., 2014. Bacterial metal resistance genes and metal bioavailability in contaminated sediments. Environmental Pollution 189, 143-151.

Shahid, M., . (2010). Citrobacter spp. simultaneously harboring blaCTX-M, blaTEM, blaSHV, blaampC, and insertion sequences IS26 and orf513: an evolutionary phenomenon of recent concern for antibiotic resistance. Journal of Clinical Microbiology 48(5), 1833.

Sutcliffe, J., Grebe, T., Tait-Kamradt, A., and Wondrack, L. (1996). Detection of erythromycin-resistant determinants by PCR. Antimicrobial Agents & Chemotherapy 40(11), 2562-2566.
