# Supplementary material for: Bacterial Heavy-Metal and Antibiotic Resistance Genes in a Copper Tailing Dam Area in Northern China
Source: Front Microbiol. 2019 Aug 20;10:1916. doi: 10.3389/fmicb.2019.01916 (PMC6710345; doi:10.3389/fmicb.2019.01916)
Supplement: Supplementary file 3 [file Data_Sheet_3.PDF]

Supplemental Material

Bacteria Resistance to Metal Pollution in a Copper Tailing Dam Area in Northern China

Jianwen Chen[1], Junjian Li[1], Hong Zhang[2], Wei Shi[1], Yong Liu[1,*]

[1] *Institute of Loess Plateau, Shanxi University, Taiyuan, Shanxi, 030006, China*
[2] *School of Environment and Resources, Shanxi University, Taiyuan 030006, China*

TABLE S3 The relative abundance of antibiotic resistance gene (ARG, in 10^{-5} copies per 16S rDNA) at each sampling site.

ARG	TD0	TD1	TD2	TD3
*tet*A	4.66 ± 2.40a	3.00 ± 2.78a	3.73 ± 3.52a	4.80 ± 2.63a
*tet*C	6.08 ± 0.95b	8.93 ± 7.36b	12.72 ± 9.81b	28.92 ± 18.53a
*tet*G	1.85 ± 0.86b	4.29 ± 1.77ab	7.21 ± 3.94a	7.78 ± 3.92a
*tet*K	0.52 ± 0.10a	0.41 ± 0.37a	0.52 ± 0.35a	0.58 ± 0.42a
*tet*A/P	0.18 ± 0.14b	1.58 ± 1.30ab	0.70 ± 0.51ab	2.03 ± 1.53a
*tet*S	22.01 ± 13.68a	20.76 ± 17.22a	11.13 ± 7.85a	20.87 ± 14.55a
*tet*X	2.92 ± 1.59b	2.68 ± 2.30b	2.23 ± 1.58b	6.12 ± 3.65a
*tet*M	0.03 ± 0.01b	0.10 ± 0.03a	0.11 ± 0.06a	0.12 ± 0.04a
*tet*O	0	0.06 ± 0.04b	0.18 ± 0.15a	0.07 ± 0.03b
*tet*Q	0.13 ± 0.12ab	0.06 ± 0.05b	0.08 ± 0.04b	0.27 ± 0.22a
*tet*W	0	0.05 ± 0.03b	0.22 ± 0.18a	0.13 ± 0.09ab
*tet*B/P	0	0.20 ± 0.09b	1.13 ± 0.85a	0.03 ± 0.02b
*ere*A	38.33 ± 7.36a	20.83 ± 12.80a	18.91 ± 7.68a	29.49 ± 16.48a
*mph*A	0.17 ± 0.08b	0.44 ± 0.29ab	0.40 ± 0.32ab	0.78 ± 0.43a
$bla_{\text{CTX-M}}$	0.02 ± 0.02a	0.02 ± 0.01a	0.02 ± 0.02a	0.03 ± 0.02a
bla_{SHV}	0.50 ± 0.24a	0.43 ± 0.30a	0.48 ± 0.34a	0.52 ± 0.31a
*sul*II	2.40 ± 0.44b	6.00 ± 3.17a	4.94 ± 2.92ab	2.78 ± 1.37b

Data are means ± standard deviation. The different letters indicate that the means are significantly different among soils ($P < 0.05$) with Duncan test. TD0 site is on the tailing dam and covered with annual herbs. TD1 site with about 15 years of poplar plantation is at the bottom of the dam. TD2 site is farmland and adjacent to TD1 site. TD3 site with about 10 years of poplar plantation is the farthest away from the dam.
