# Supplementary material for: Bacterial Heavy-Metal and Antibiotic Resistance Genes in a Copper Tailing Dam Area in Northern China
Source: Front Microbiol. 2019 Aug 20;10:1916. doi: 10.3389/fmicb.2019.01916 (PMC6710345; doi:10.3389/fmicb.2019.01916)
Supplement: Supplementary file 4 [file Data_Sheet_4.PDF]

Supplemental Material

Bacteria Resistance to Metal Pollution in a Copper Tailing Dam Area in Northern China

Jianwen Chen[1], Junjian Li[1], Hong Zhang[2], Wei Shi[1], Yong Liu[1,*]

[1] *Institute of Loess Plateau, Shanxi University, Taiyuan, Shanxi, 030006, China*
[2] *School of Environment and Resources, Shanxi University, Taiyuan 030006, China*

TABLE S4 The Spearman correlations among heavy metals and the relative abundance of metal resistant genes.

	As	Cd	Cr	Cu	Ni	Pb	Zn
*pco*A	ns	ns	ns	ns	ns	ns	ns
*cop*A	ns	ns	ns	ns	ns	-*	ns
*cop*B	-**	ns	ns	-**	ns	ns	ns
*czc*C	*	*	ns	**	ns	ns	ns
*czc*D	ns	**	ns	*	ns	**	ns
*czc*A	ns	**	ns	*	ns	ns	ns
*pbr*T	*	**	ns	ns	ns	ns	ns
*chr*B	ns	*	ns	ns	ns	**	ns
*ars*B	**	**	ns	**	ns	ns	ns
*ars*C	*	**	ns	*	ns	ns	ns
MRGs	*	**	*	*	ns	ns	ns

** Correlation is significant at the 0.01 level (2-tailed). * Correlation is significant at the 0.05 level (2-tailed). ns means not significant correlation at the 0.05 level (2-tailed). “-” means negative correlations.
