# Supplementary material for: Bacterial Heavy-Metal and Antibiotic Resistance Genes in a Copper Tailing Dam Area in Northern China
Source: Front Microbiol. 2019 Aug 20;10:1916. doi: 10.3389/fmicb.2019.01916 (PMC6710345; doi:10.3389/fmicb.2019.01916)
Supplement: Supplementary file 5 [file Data_Sheet_5.PDF]

Supplemental Material

Bacteria Resistance to Metal Pollution in a Copper Tailing Dam Area in Northern China

Jianwen Chen[1], Junjian Li[1], Hong Zhang[2], Wei Shi[1], Yong Liu[1,*]

[1] *Institute of Loess Plateau, Shanxi University, Taiyuan, Shanxi, 030006, China*
[2] *School of Environment and Resources, Shanxi University, Taiyuan 030006, China*

TABLE S5 The Spearman correlations among heavy metals and the relative abundance of antibiotic resistant genes.

	As	Cd	Cr	Cu	Ni	Pb	Zn
*tet*A	ns	ns	ns	ns	ns	-**	ns
*tet*C	ns	ns	ns	ns	ns	ns	ns
*tet*G	ns	ns	ns	ns	ns	ns	ns
*tet*K	ns	ns	ns	ns	ns	-**	ns
*tet*A/P	*	ns	ns	ns	ns	ns	ns
*tet*S	ns	ns	ns	ns	ns	-**	-**
*tet*X	ns	ns	ns	ns	ns	-**	ns
*tet*M	ns	ns	*	ns	ns	ns	ns
*tet*O	ns	ns	ns	ns	ns	ns	ns
*tet*Q	ns	ns	ns	ns	ns	-*	ns
*tet*W	ns	ns	ns	*	ns	ns	*
*tet*B/P	ns	ns	*	*	ns	ns	*
*ere*A	-*	ns	ns	ns	ns	-*	ns
*mph*A	ns	ns	ns	ns	ns	ns	ns
$bla_{\text{CTX-M}}$	ns	ns	ns	ns	ns	-**	ns
bla_{SHV}	ns	ns	ns	ns	ns	-**	ns
*sul*II	ns	**	ns	ns	ns	*	*
*int*I1	ns	ns	ns	ns	ns	ns	ns
*tnp*A	ns	ns	ns	*	ns	-**	ns
ARGs	ns	ns	ns	ns	ns	ns	ns

** Correlation is significant at the 0.01 level (2-tailed). * Correlation is significant at the 0.05 level (2-tailed). ns means not significant correlation at the 0.05 level (2-tailed). “-” means negative correlations.
